# Higher plasma cystatin C is associated with mortality after acute respiratory distress syndrome: findings from a Fluid and Catheter Treatment Trial (FACTT) substudy

**DOI:** 10.1186/s13054-020-03111-1

**Published:** 2020-07-11

**Authors:** Carolyn M. Hendrickson, Yuenting D. Kwong, Annika G. Belzer, Michael G. Shlipak, Michael A. Matthay, Kathleen D. Liu

**Affiliations:** 1 Division of Pulmonary and Critical Care Medicine, Department of Medicine, Zuckerberg San Francisco General Hospital and Trauma Center, University of California San Francisco, 1001 Potrero Ave, San Francisco, CA 94110 USA; 2 Division of Nephrology, Department of Medicine, University of California San Francisco, San Francisco, CA USA; 3 Yale School of Medicine, New Haven, CT USA; 4 Division of General Internal Medicine, San Francisco VA Medical Center, San Francisco, CA USA; 5 Kidney Health Research Collaborative, San Francisco VA Medical Center, University of California, San Francisco, CA USA; 6 Department of Medicine, Cardiovascular Research Institute, University of California, San Francisco, CA USA; 7 Department of Anesthesia, Cardiovascular Research Institute, University of California, San Francisco, CA USA

**Keywords:** Cystatin C, Acute respiratory distress syndrome (ARDS), Acute kidney injury (AKI)

## Abstract

**Background:**

Cystatin C is a well-validated marker of glomerular filtration rate in chronic kidney disease. Higher plasma concentrations of cystatin C are associated with worse clinical outcomes in heterogenous populations of critically ill patients and may be superior to creatinine in identifying kidney injury in critically ill patients. We hypothesized that elevated levels of plasma cystatin C in patients with acute respiratory distress syndrome (ARDS) would be associated with mortality risk.

**Methods:**

In a retrospective study, cystatin C was measured by nephelometry on plasma obtained at enrollment from 919 patients in the Fluid and Catheter Treatment Trial. Multivariable logistic regression was performed testing the association between quartiles of cystatin C and 60-day mortality. Analyses were stratified by acute kidney injury (AKI) status identified in the first 7 days after enrollment by Kidney Disease: Improving Global Outcomes (KDIGO) criteria.

**Results:**

Cystatin C was significantly higher among those patients who died compared to those who survived to 60 days [1.2 (0.9–1.9) mg/L vs. 0.8 (0.6–1.2) mg/L, *p* < 0.001]. Compared to the lower three quartiles, subjects in the highest quartile of cystatin C had a significantly higher odds of death at 60 days [OR 1.8 (1.2–2.6), *p* = 0.003 in adjusted analyses]; the odds of death incrementally rose in higher cystatin C quartiles compared to the lowest quartile (OR 1.1, 1.8, and 2.5). In adjusted analyses stratified by AKI status, compared to subjects in the lower three quartiles, subjects in the highest quartile of cystatin C with AKI had a significantly higher odds of death at 60 days both in participants with AKI [OR 1.6 (1.0–2.4), *p* = 0.048] and those without AKI [OR 2.4 (1.2–5.0), *p* = 0.017]. In adjusted analyses, there was no significant association between sex-stratified baseline creatinine quartiles and mortality.

**Conclusions:**

Higher plasma levels of cystatin C on enrollment were strongly associated with mortality at 60 days in patients with ARDS with and without AKI identified by creatinine-based definitions. Compared to creatinine, cystatin C may be a better biomarker of kidney function in patients with ARDS and therefore identify patients with multiple organ failure at higher risk of death.

## Background

Identifying patients who have the highest risk of death after the acute respiratory distress syndrome (ARDS) and understanding the biology driving this risk are important to both clinicians and researchers. Studying the predictive value of biomarkers measured early in the course of ARDS may help with risk stratification for important clinical outcomes including death and multiple organ dysfunction after ARDS. Acute kidney injury (AKI) has been associated with increased mortality among critically ill patients, and the development of AKI after ARDS by definition marks the development of multiple organ dysfunction [1–4]. Cystatin C is a 13-kDa inhibitor of cysteine proteases and a housekeeping gene expressed in all nucleated cells at a steady rate. Because of its small size and basic pH, this molecule is freely filtered at the glomerulus, then reabsorbed and fully catabolized, but not secreted by the proximal renal tubule. These properties make cystatin C an ideal marker for glomerular filtration rate (GFR). Cystatin C is a well-validated marker of kidney function in chronic kidney disease and may be a superior marker of acute kidney injury leading to impaired GFR compared to serum creatinine among critically ill patients [5, 6]. Furthermore, plasma cystatin C measurements are clinically available in many institutions. Elevated cystatin C is associated with higher mortality in heterogenous cohorts of critically ill patients [5, 7, 8], but this finding has not been studied in a large cohort of patients with ARDS.

In this retrospective cohort study, we measured cystatin C in plasma samples obtained from 919 subjects with ARDS on enrollment in the Fluid and Catheter Treatment Trial (FACTT) [9]. Using adjusted logistic regression models, we tested the association between plasma cystatin C and 60-day mortality. We hypothesized that plasma cystatin C measured early in the course of ARDS would identify a subset of the most severely ill patients and that this biomarker would add predictive and biological information to mortality prediction models in ARDS above and beyond identification of AKI cases using creatinine-based definitions.

## Methods

The ARDS Network FACTT trial is a large randomized controlled trial with a factorial design comparing a fluid conservative to a fluid liberal management strategy and comparing the use of pulmonary artery catheters to central venous catheters in the management of 1000 patients with ARDS [9]. Subjects were enrolled within 48 h of developing ARDS, and patients with end-stage renal disease or requiring renal replacement therapy were excluded from the study. For this retrospective cohort study, 919 plasma samples were available for cystatin C measurement, which were made on a Dade-Behring BNII nephelometer. Estimated glomerular filtration rate (eGFR) was not reported in this cohort because these estimates would be unreliable given the available plasma creatinine and cystatin C measurements were not made at steady state. Sex-stratified multivariate models adjusted for baseline creatinine were performed separately from the analyses stratified by AKI, which are the main focus of our analysis. AKI was identified by applying Kidney Disease: Improving Global Outcomes (KDIGO) criteria to all available creatinine (Cr) measurements in the first 7 days after study enrollment. AKI cases were identified as an increase in Cr ≥ 0.3 mg/dL over 48 h, to levels greater than or equal to 1.5 times baseline Cr or dialysis initiation within 7 days. Baseline Cr was defined by serum Cr at study enrollment. We repeated adjusted and stratified analyses after reclassifying AKI cases by accounting for the effect of fluid balance on the volume of distribution of creatinine [10]. Previous work using latent class analysis (LCA) has identified subphenotypes with different mortality rates and differential response to therapy within large randomized controlled trials of patients with ARDS, including FACTT. We adjusted for these subphenotypes which have also been characterized as hypoinflammatory or subphenotype 1, and hyperinflammatory or subphenotype 2 [11, 12]. All variables considered for inclusion in the multivariable logistic regression models were examined for distribution and missingness, and appropriate model checking was performed. Multiple imputation was used to address 4% missing data for the APACHE III variable. Logistic regression models were used to test the association between cystatin C and mortality adjusted for important confounders, and the post-estimation area under the receiver operating curve (AUROC) was calculated. Because the linearity assumption of the logistic regression models was violated when considering cystatin C as a continuous variable, even when testing several options for transformation of the independent variable, including the commonly used inverse function, cystatin C was analyzed by quartiles defined using data from the full cohort. Cystatin C was divided into quartiles at the following cut points: quartile 1 (0.2–0.67 mg/L), quartile 2 (0.68–0.90 mg/L), quartile 3 (0.91–1.37 mg/L), and quartile 4 (1.38–5.2 mg/L). The same quartile cutoffs from cystatin C measurements in the full cohort were used in all analyses including stratified analyses. Post-estimation assessments of discrimination and calibration were performed using standard assessments with the *C* statistic and Hosmer-Lemeshow goodness of fit test. Results from a post-estimation linear test for trend are reported to describe the association between cystatin C quartiles and mortality in adjusted analyses. Sensitivity analyses were performed excluding subjects with clinical and demographic characteristics that are believed to influence plasma cystatin C levels and possibly act as confounders of the association between cystatin C and mortality, including cancer, trauma, and recent surgery. Furthermore, likelihood ratio testing was used to eliminate variables from models that were adjusted for a variety of other factors known to affect cystatin C levels. When compared to the parsimonious final models presented here, the models with additional variables did not improve the model fit. The following variables were tested and eliminated from the final model: body mass index (BMI), diabetes, baseline white blood cell count (WBC), serum albumin, and a history of cardiovascular disease. All analyses were performed using STATA version 15 (StataCorp, College Station, TX).

## Results

**Fig. 1:**
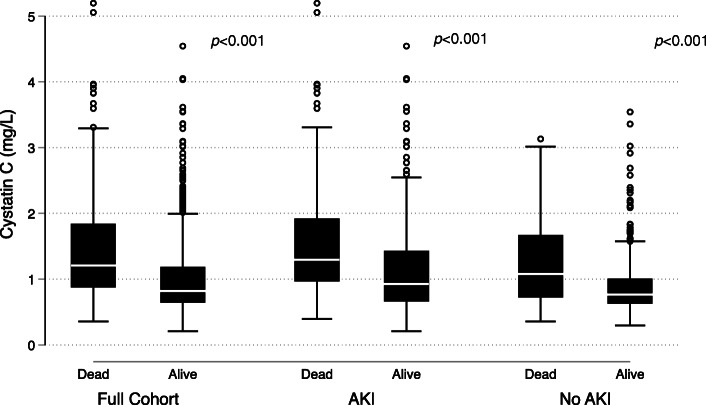
Box and whisker plots of the raw plasma cystatin C data in the full cohort and stratified by Kidney Disease: Improving Global Outcomes (KDIGO) acute kidney injury (AKI) status show that cystatin C is higher among those who died by 60 days compared to those who survived. This difference was statistically significant by Wilcoxon’s rank-sum testing in the full cohort and both strata of AKI status. The cystatin C quartile cutoffs were established in the full cohort and applied to the data stratified by AKI status

The demographic and clinical characteristics of the 919 subjects included in this analysis of the FACTT study are displayed in Table 1. The median age of subjects was 49 years, and 53% of subjects were female. The mortality rate was 28%. The incidence of AKI in the full cohort was 53% without adjusting for fluid balance and 61% after adjusting for fluid balance. The median APACHE III score was 91 (IQR 70–117). The most common primary risk factor for ARDS was pneumonia (426 subjects, 46%) followed by sepsis (218 subjects, 24%). A total of 394 subjects (43%) had sepsis listed as either a primary or a secondary risk factor for ARDS. The median baseline creatinine value measured in the full cohort was 1.0 mg/dL (IQR 0.7–1.5 mg/dL). The median cystatin C level at enrollment was 0.9 mg/L, and the interquartile range was 0.7–1.4 mg/L. Plasma cystatin C levels were higher among those in the hyperinflammatory subphenotype compared with those in the hypoinflammatory subphenotype [1.3 (0.9–2.1) mg/L vs. 0.8 (0.6–1.1) mg/L, *p* value< 0.0001]. Cystatin C was higher among those who died compared to those who survived [1.2 (0.9–1.9) mg/L vs. 0.8 (0.6–1.2) mg/L, *p* < 0.001] (Table 1). This difference remained statistically significant when the cohort was stratified by AKI status (Fig. 1). In subjects with AKI, cystatin C was higher among those who died compared to those who survived [1.3(1.0–1.9) mg/L vs. 0.9 (0.7–1.4) mg/L, *p* < 0.0001], and the same pattern was observed in subjects without AKI [1.1 (0.7–1.7) mg/L vs. 0.8 (0.6–1.0) mg/L, *p* < 0.0001]. As has been previously reported in a different subgroup analysis of the FACTT trial [12], baseline serum creatinine was higher among subjects in subphenotype 2 compared to subphenotype 1 [1.6 (1.1–2.4) vs. 0.9 (0.7–1.2), *p* < 0.0001].

**Table 1:** Demographics, clinical Characteristics, and biomarkers by 60-day mortality

	Full cohort, *n* = 919	Alive at 60 days, *n* = 658	Dead by 60 days, *n* = 261	*p* value
Age	49 (38–61)	47 (37–57)	57 (41–70)	**< 0.001**
Female sex	489 (53)	338 (52)	151 (58)	0.076
BMI	27 (23–32)	28 (24–33)	26 (22–31)	**0.005**
Race/ethnicity				
White	602 (66)	459 (70)	143 (55)	**< 0.001**
Black	193 (21)	121 (18)	72 (28)	
Hispanic	124 (13)	78 (12)	46 (18)	
Fluid conservative arm	467 (51)	342 (52)	125 (48)	
Baseline WBC (10^3^/μL)	11.8 (7.2–17.1)	12.0 (7.8–17.1)	10.6 (5.8–17.3)	**0.046**
Baseline creatinine (mg/dL)	1.0 (0.7–1.5)	0.8 (0.7–1.4)	1.2 (0.9–1.8)	**< 0.001**
Cystatin C (mg/L)	0.9 (0.7–1.4)	0.8 (0.6–1.2)	1.2 (0.9–1.9)	**< 0.001**
AKI^#^				
Actual	486 (53)	306 (47)	180 (69)	**< 0.001**
Adjusted for fluid balance	559 (61)	348 (53)	211 (81)	**< 0.001**
APACHE III score	91 (70–117)	85 (65–104)	114 (91–133)	**< 0.001**
Primary ARDS risk factor				
Pneumonia	426 (46)	303 (46)	123 (47)	**0.002**
Sepsis	218 (24)	138 (21)	80 (31)	
Aspiration	138 (15)	104 (16)	34 (13)	
Trauma	71 (8)	62 (9)	9 (3)	
Multiple transfusion	9 (1)	7 (1)	2 (1)	
Other	57 (6)	44 (7)	13 (5)	
Hyperinflammatory LCA subphenotype^⍺^	252 (27)	137 (54)	115 (46)	**< 0.001**
Comorbidities				
Solid tumor	14 (2)	8 (1)	6 (2)	0.25
Lymphoma	13 (1)	4 (1)	9 (4)	**0.001**
Leukemia	20 (3)	9 (1)	11 (4)	**0.009**
Recent surgery	46 (5)	37 (6)	9 (3)	0.17

Data presented as *n* (%) or median (IQR)

*p* value refers to a comparison of those survivors to those who died using rank-sum, Pearson’s chi^2^, or Fisher’s exact tests as appropriate

*BMI* body mass index, *WBC* white blood cell count, *AKI* acute kidney injury by KDIGO criteria, *IQR* interquartile range

^#^AKI by Kidney Disease: Improving Global Outcomes (KDIGO) criteria

^⍺^*LCA* latent class analysis, Famous et al. [12]

**Fig. 2:**
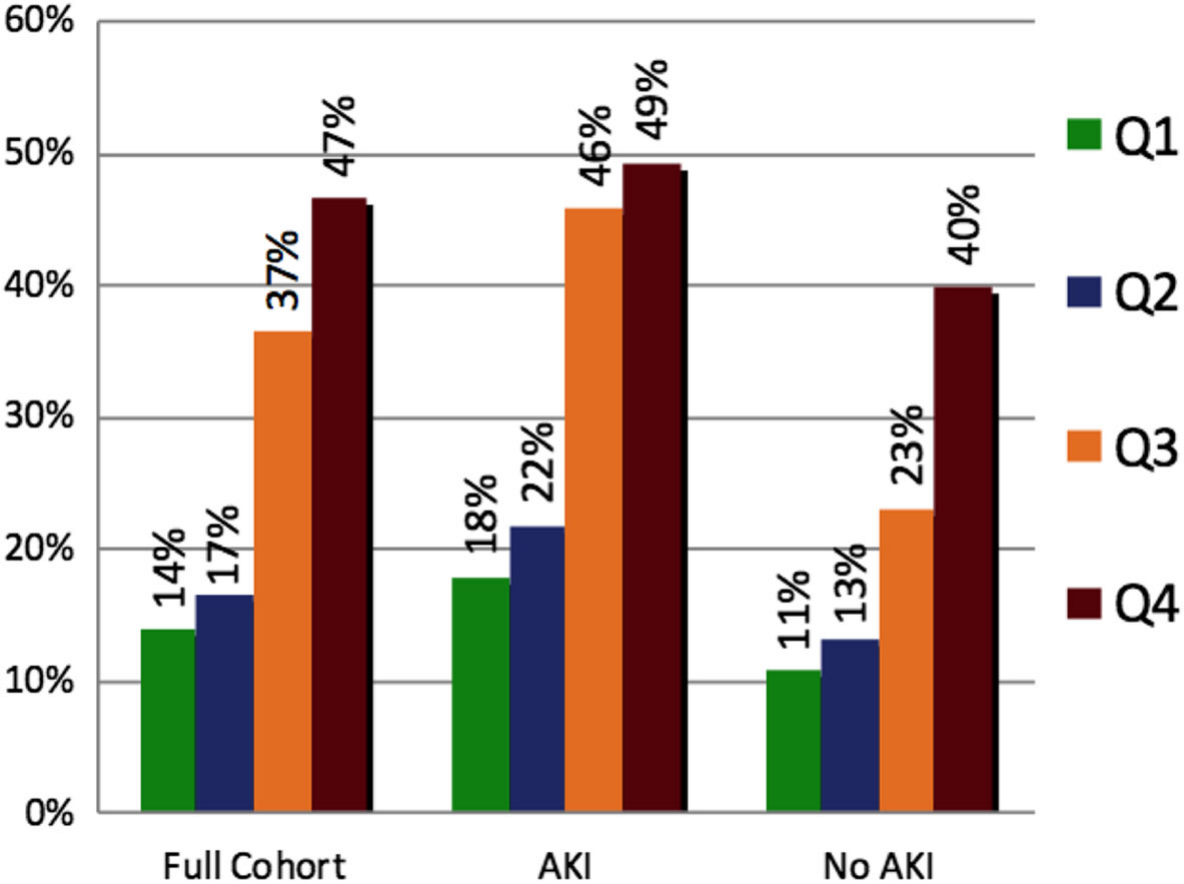
Bar graphs of the crude 60-day mortality rate by plasma cystatin C quartile in the full cohort and in the strata defined by acute kidney injury (AKI) by Kidney Disease: Improving Global Outcomes (KDIGO) criteria. The cystatin C quartile cutoffs were established in the full cohort and applied to the data stratified by AKI status. Crude mortality rates are similar in each quartile between the full cohort and the stratified data

The crude 60-day mortality rate by cystatin C quartile is shown in Fig. 2. The highest quartile of cystatin in the full cohort had an elevated mortality rate that was observed in both AKI strata. Even among subjects without AKI by creatinine-based definitions, the 60-day mortality rate in the highest quartile of cystatin C was 40%.

In the full cohort, a multivariate logistic regression model adjusted for sepsis, age, sex, APACHE III score, AKI status, treatment arm, and LCA subphenotype showed that higher quartiles of cystatin C were strongly associated with 60-day mortality. In contrast, in adjusted analyses, there was no significant association between sex-stratified baseline creatinine quartiles and mortality (Table 2). In the full cohort, subjects in the highest quartile of cystatin C had a more than the twofold odds of death compared to those in the lowest quartile [OR 2.5 (1.5–4.2); linear test for trend *p* = 0.002] (Table 4) and compared to subjects in the lower three quartiles combined [OR 1.8 (1.2–2.6, *p* = 0.003)] (Table 3). The relationship between cystatin C and 60-day mortality in adjusted models was not significantly different between subjects with and without AKI. Post-estimation tests for linear trend for the association between cystatin C quartiles and mortality were significant in the full cohort and among subjects with AKI, but not among subjects without AKI (Table 4). However, there was no statistically significant interaction between AKI status and cystatin C in adjusted models. Additionally, there was no significant interaction between LCA subphenotype and cystatin C in the full cohort or in the analyses stratified by AKI status. The AUROC for the full multivariate model of 60-day mortality was 0.79. The AUROCs for univariate models of 60-day mortality including APACHE III and plasma cystatin C were 0.74 and 0.68 respectively. In a bivariate model including APACHE III and plasma cystatin C, the AUROC was 0.74.

**Table 2:** No association between sex-stratified baseline creatinine quartiles and 60-day mortality in adjusted logistic regression models

		OR (95% CI)	*p* value
**Female sex** (*n* = 489) covariates: sepsis, age, sex, APACHE III*, treatment arm, LCA subphenotype^⍺^			
Baseline creatinine	Q2	0.6 (0.3–1.3)	0.67
	Q3	0.8 (0.4–1.5)	
	Q4	1.1 (0.6–2.2)	
**Male sex** (*n* = 430) covariates: sepsis, age, sex, APACHE III*, treatment arm, LCA subphenotype^⍺^			
Baseline creatinine	Q2	1.4 (0.7–2.8)	0.26
	Q3	1.9 (0.9–3.8)	
	Q4	2.1 (1.0–4.4)	

All analyses compare stated quartile to first quartile (Q1)

*p* value refers to post-estimation global test for the null hypothesis that creatinine quartiles are not associated with death

*APACHE III scores exclude renal variables in these models with creatinine as predictor

^⍺^*LCA* latent class analysis, Famous et al. [12]

**Table 3:** Higher cystatin C is associated with 60-day mortality in subjects with and without AKI

	OR (95% CI)	*p* value
**Full cohort** (*n* = 919)		
Cystatin C Q4	1.8 (1.2–2.6)	0.003
**With acute kidney injury** (*n* = 486)		
Cystatin C Q4	1.6 (1.0–2.4)	0.048
**Without acute kidney injury** (*n* = 433)		
Cystatin C Q4	2.4 (1.2–5.0)	0.017

All analyses compare the highest quartile to the lower three quartiles

Q4—highest quartile of cystatin C with quartiles determined by ranges of cystatin C in full cohort

All models adjusted for sepsis, age, sex, APACHE III, treatment arm, and LCA subphenotype. Full cohort model also adjusted for acute kidney injury (AKI) by Kidney Disease: Improving Global Outcomes (KDIGO) creatinine-based definition

**Table 4:** The adjusted association between 60-day mortality and cystatin C overall and stratified by AKI status

		OR (95% CI)	*p* value
**Full cohort** (*n* = 919) covariates: sepsis, age, sex, APACHE III, AKI, treatment arm, LCA subphenotype^⍺^			
Cystatin C	Q2	1.1 (0.6–1.8)	0.0002
	Q3	1.8 (1.1–3.1)	
	Q4	2.5 (1.5–4.2)	
**With acute kidney injury** (*n* = 486) covariates: sepsis, age, sex, APACHE III, treatment arm, LCA subphenotype^⍺^			
Cystatin C	Q2	1.2 (0.6–2.6)	0.0032
	Q3	2.6 (1.3–4.9)	
	Q4	2.7 (1.4–5.3)	
**Without acute kidney injury** (*n* = 433) covariates: sepsis, age, sex, APACHE III, treatment arm, LCA subphenotype^⍺^			
Cystatin C	Q2	0.7 (0.3–1.6)	0.094
	Q3	0.9 (0.4–2.2)	
	Q4	2.0 (0.8–5.2)	

*p* value refers to post-estimation linear test for trend across cystatin C quartiles

*AKI* acute kidney injury by Kidney Disease: Improving Global Outcomes (KDIGO) creatinine-based definition

^⍺^*LCA* latent class analysis, Famous et al. [12]

All analyses compare stated quartile to first quartile (Q1)

**Fig. 3:**
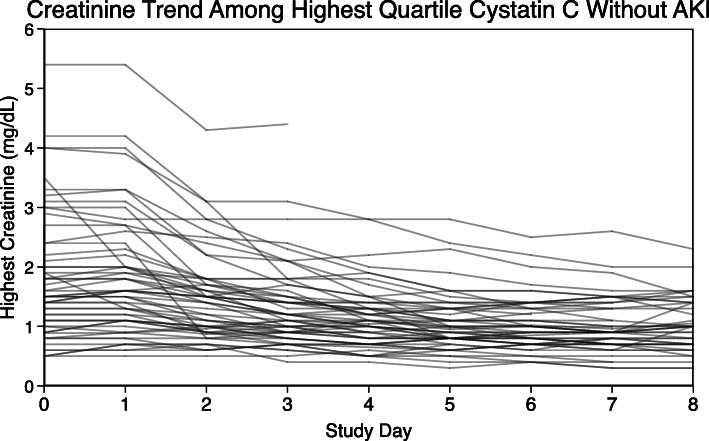
Creatinine trends plotted for each of the 65 subjects in the highest quartile of plasma cystatin C that did have acute kidney injury (AKI) by Kidney Disease: Improving Global Outcomes (KDIGO) criteria show that the creatinine trajectories for these individuals are down-trending or plateaued in the first 8 days after enrollment

The subjects in the highest quartile of cystatin C who did not meet the creatinine-based definition of AKI (*n* = 65) had a substantially elevated risk of death compared to subjects without AKI in the lower three quartiles of cystatin C [OR 2.4 (1.2–5.0, *p* = 0.017)] (Table 3). These subjects were of particular interest in this analysis. We examined the clinical and demographic characteristics of this group in an effort to understand the drivers of the observed statistical association (Table 5). The median age of these subjects was 58, and 31% were female. The creatinine trends over the first 8 study days among subjects with the highest quartile of cystatin C but no AKI are plotted in Fig. 3. These data show that the daily creatinine recorded among these individuals was either down-trending or stable over time. The median value of baseline creatinine on study enrollment among these 65 patients was 1.5 mg/dL (IQR 1.1–2.2 mg/dL). Only 10 of these 65 subjects died before study day 9, and the median survival time among those who died was 16 days.

**Table 5:** Characteristics of 65 subjects without acute kidney injury in the highest quartile of cystatin C

Age (years)	58 (46–69)
Female sex	20 (31%)
BMI	28.1 (24.2–37.1)
Baseline serum creatinine (mg/dL)	1.5 (1.1–2.2)
Baseline serum creatinine > 1.5 mg/dL	32 (49%)
Cystatin C (mg/L)	1.8 (1.6–2.3)
Mortality at 60 days	26 (40%)
Survival time to 60 days (days)	60 (23–60)
Died before study day 8	10 (15%)
Survival time among those who died (days)	16 (1–33)

Acute kidney injury defined by Kidney Disease: Improving Global Outcomes (KDIGO) creatinine-based definition

*BMI* body mass index

Data presented as *n* (%) or median (IQR)

## Discussion

Here, we have found for the first time that higher plasma cystatin C concentrations measured early in the course of ARDS are associated with higher mortality and that this association persists after adjustment for AKI defined by creatinine-based criteria. Earlier studies reported that elevated cystatin C is associated with higher mortality in heterogenous cohorts of critically ill patients [5, 7, 8], but this finding had not previously been validated in a large cohort of patients with ARDS or studied among critically ill patients cared for in North America.

The association between elevated plasma cystatin C and death after ARDS is most likely driven by the glomerular filtration rate pathway, capturing kidney dysfunction in a way not captured by other measures of illness severity. This dysfunction could be either acute, chronic, or both. Plasma cystatin C may identify additional patients with AKI and therefore multiorgan failure who do not meet creatinine-based definitions of AKI. In this cohort of patient with ARDS, the highest quartile of cystatin C measurements identified 65 individuals, 7% of the full cohort, with likely kidney dysfunction not detected by creatinine-based definitions of AKI (Table 5, Fig. 3). In other critically ill populations, cystatin C appears to be a superior marker of glomerular filtration rate than creatinine [5, 6, 13]. If cystatin C is a more sensitive marker of AKI than creatinine among those with ARDS, it can accurately identify subjects with multiorgan failure, a well-established risk factor for death among critically ill patients [14–19]. Additionally, cystatin C is a more sensitive marker of chronic kidney disease (CKD) than creatinine in many populations and CKD is a known risk factor for death after critical illness [20, 21]. While it is possible that the association between cystatin C and mortality after ARDS is working through a non-glomerular filtration rate pathway, this explanation is purely speculative. In sensitivity analyses excluding subjects with available data on conditions known to increase cystatin C production, we did not find any evidence supporting this hypothesis. Although we are unable to test the mechanism of association between elevated cystatin C, multivariable models adjusted for important potential confounders or mediators including APACHE III score and LCA subphenotype showed a robust association between cystatin C and mortality among patients with ARDS. We conclude that this biomarker provides valuable prognostic information not otherwise captured by established markers of critical illness severity.

The 65 subjects in the highest quartile of cystatin C but without AKI by creatinine-based definitions were of particular interest. Studying basic demographic assessments of age, sex, and BMI gave no indication that these individuals may have had lower muscle mass that would make creatinine a less-reliable marker of GFR. The data presented in Table 5 and Fig. 2 do not clearly suggest that these 65 patients were simply patients with previously undiagnosed chronic kidney disease, but with the available information, we cannot rule out that possibility. Only 10 of these 65 subjects died before study day 9, and the median survival time among those who died was 16 days. These data suggest that survival bias censoring the trajectory of creatinine values does not explain why this group of subjects with high cystatin C did not have AKI by a creatinine-based definition. Taken together, these data suggest that this group of study subjects was not classified as having AKI because they were most likely on the downward trajectory or plateau phase of their creatinine measurements, and the AKI occurred earlier in their course of illness before study enrollment in FACTT.

In this secondary data analysis, a single measurement of plasma cystatin C early in the course of ARDS provides prognostic information about mortality beyond creatinine and creatinine-based definitions of AKI and this may be an appealing biomarker to measure for both research and clinical care purposes. Plasma cystatin C measurement is widely available in many clinical settings and may allow clinicians to identify patients with ARDS at highest risk of death, early in their course of illness. Interestingly, cystatin C differed between LCA subphenotypes. Furthermore, it appears to provide additional information beyond both APACHE III score and LCA subphenotype and therefore may be of interest in future clinical research studies focused on enhancing enrollment of subjects at highest risk of death or studies using risk stratification to assign or evaluate treatment interventions. Specifically, more work is needed to understand the relationship between cystatin C and LCA subphenotypes in ARDS.

This study has several strengths. The FACTT study enrolled a large number of patients with well-adjudicated ARDS. Detailed data collection allowed for the rigorous adjudication of AKI by KDIGO criteria and adjustment for APACHE III score and sepsis in multivariable analyses. Prior work using clinical data and biomarkers measured in patients enrolled in the FACTT trial identified subphenotype latent classes with differential response to therapy. We included adjustment for subphenotypes in our models to strengthen the importance of this novel finding of the association between cystatin C and mortality after ARDS. Our study has some limitations. The retrospective design of this study does not allow us to test the mechanisms driving the association between cystatin C and mortality after ARDS. Nor do these data allow us to test for differential associations between cystatin C and death after ARDS in patients with acute kidney injury from different causes. As these plasma biomarkers are not in steady state during critical illness, timing of creatinine and cystatin C measurements in this cohort do not allow for meaningful estimation of glomerular filtration rate or accurate classification of chronic kidney disease prior to study enrollment.

## Conclusions

The strong association between mortality and elevated plasma cystatin C measured early in the course of ARDS was robust to adjustment for many important confounders or potential mediators, and this association persists after adjustment for AKI defined by creatinine-based criteria. Among patients with ARDS, cystatin C may identify kidney dysfunction and multiple organ failures that increase the risk of death and are not captured by other commonly measured assessments of severity of illness. These findings are likely to be of interest to a broad audience of both clinicians and investigators who are designing clinical trials.

## Data Availability

The parent FACTT data is available from the National Heart, Lung and Blood Institute. If requested, the investigators will work with the National Heart, Lung and Blood Institute and the requestors to make the cystatin C measurements available.

## Abbreviations

ARDS: Acute respiratory distress syndrome
AKI: Acute kidney injury
FACTT: Fluid and Catheter Treatment Trial
KDIGO: Kidney Disease: Improving Global Outcomes
LCA: Latent class analysis
